# Correcting gut dysbiosis can ameliorate inflammation and promote remyelination in multiple sclerosis – No

**DOI:** 10.1177/13524585211016722

**Published:** 2021-05-28

**Authors:** Christopher E McMurran

**Affiliations:** Department of Clinical Neurosciences, University of Cambridge, Cambridge, UK

It is now well established, at least in animal models, that pathological changes in the gut microbiota (dysbiosis) can influence inflammation in the central nervous system (CNS). The idea of a disease-modifying therapy for MS that acts through the microbiota, safely and relatively non-invasively, is certainly attractive; and progress is ongoing towards immunomodulatory treatments of this nature. However, the proposal that the same approach can promote remyelination in people with MS is not supported by current evidence.

I will address this proposal in three parts:

1. Can correcting gut dysbiosis ameliorate inflammation?
2. Can this promote remyelination?
3. Does this apply to people with MS?

## Can correcting gut dysbiosis ameliorate inflammation?

In short: yes. Over the past decade, -omics approaches have revealed reproducible taxa-level differences between the gut microbiota of people with MS and healthy controls.^1^ Meanwhile, gnotobiotic animal models have yielded convincing evidence for a causal role of dysbiosis in MS pathogenesis. For example, Berer et al.^2^ demonstrated how faecal microbial transplantation (FMT) from an identical twin with MS can trigger CNS autoimmunity in mice much more reliably than colonisation from their unaffected twin. In turn, a variety of interventions that target the microbiota have been shown to ameliorate inflammation and disease severity in animal models of MS – including antibiotics, probiotics, prebiotics and FMT (reviewed^3^).

## Can these changes promote remyelination?

A coordinated inflammatory response removes myelin debris and provides growth factors to encourage remyelination, so there is a theoretical basis for changes in the microbiota to influence remyelination via neuroinflammation. We tested this hypothesis in germ-free (GF), antibiotics-treated and probiotic-treated mice.^4^ While these three interventions all produced changes in the inflammatory response to toxin-induced demyelination, they had no convincing impact on remyelination itself. Broad-spectrum antibiotics caused a delay in oligodendrocyte progenitor cell (OPC) differentiation; however, this finding was not reversed by FMT and, alongside the negative data from other models, it likely represented off-target effects of the antibiotic regime. Notably, in the complete absence of a microbiota, GF mice regenerated myelin to the same extent and with the same ultrastructural appearance as control mice.

Other work interrogating the timing of microbial depletion in a different model, spontaneous experimental autoimmune encephalomyelitis (EAE), gives a consistent picture.^5^ Prophylactic antibiotic treatment before disease onset protected the mice from developing EAE but giving antibiotics in established disease had no clinical effect. This finding is further evidence that, while the microbiota is key to the induction of CNS autoimmunity, it then becomes a relatively minor player during ongoing damage and repair.

We observed an uncoupling of remyelination from changes in the innate immune response,^4^ but there are other hypothetical means for the microbiota to influence remyelination besides neuroinflammation. Gut microbial metabolites such as butyrate^6^ and p-cresol^7^ can signal directly to oligodendrocyte progenitor cells in vitro and are respectively positive and negative regulators of their differentiation to oligodendrocytes. However, at present there is no evidence to show that such metabolites, when produced by the microbiota in vivo, have a physiological effect on remyelination.

## How do these results apply to people with MS?

Several clinical trials are ongoing to test microbiota-based interventions in MS, but there is little published literature at the time of writing. Most studies focus on changes in the peripheral immune response, which is in more direct contact with the microbiota and easily assessed through blood tests. For example, Tankou et al.^8^ administered a probiotic to 9 relapsing-remitting MS patients and 13 healthy controls, demonstrating anti-inflammatory changes in blood monocytes and dendritic cells. However, the question as to whether these interventions lead to amelioration of CNS inflammation can only be answered through MRI outcomes or clinical relapses. A single randomised control trial (*N* = 60) showed an improvement in expanded disability status scale (EDSS) among patients receiving a probiotic.^9^ Results from further studies using imaging and clinical outcome measures are awaited, to build upon this encouraging outcome.

No study has directly addressed whether similar interventions could promote remyelination in people. However, such a trial would be low priority given the negative preclinical data, combined with the added challenges of measuring remyelination in human subjects. Whereas the microbiota’s effects on neuroinflammation have a strong basis in rodent models, the preclinical evidence argues *against* the microbiota being an important factor in remyelination. It therefore seems unlikely that correcting dysbiosis would give a meaningful signal in patients, among additional experimental noise not seen in laboratory animals – such as genetic diversity, lifestyle and concomitant medications. With the field of remyelination-promoting clinical therapies in its infancy, it would be prudent to focus on interventions that have the strongest experimental basis.^10^

In summary, the preclinical evidence shows that remedying a dysfunctional microbiota can improve inflammation – but not promote remyelination – in animal models of demyelination. For people with MS, current trials mainly focus on the peripheral immune response and studies with imaging outcomes are awaited. The question of whether correcting dysbiosis could promote remyelination in MS has not been studied directly, but this seems far-fetched in the context of the evidence to date.

As the literature linking the microbiota to host physiology has expanded in recent years, ‘correcting gut dysbiosis’ is sometimes touted as a panacea among academics and the public alike. However, as with any biological system, there will be limits to what the microbiota can achieve. While the idea is enticing, the promotion of remyelination for patients with MS most likely lies beyond these limits.
